# An Orphan Chemotaxis Sensor Regulates Virulence and Antibiotic Tolerance in the Human Pathogen *Pseudomonas aeruginosa*

**DOI:** 10.1371/journal.pone.0042205

**Published:** 2012-08-01

**Authors:** Heather Pearl McLaughlin, Delphine L. Caly, Yvonne McCarthy, Robert Patrick Ryan, John Maxwell Dow

**Affiliations:** BIOMERIT Research Centre, Department of Microbiology, University College Cork, Cork, Ireland; The Scripps Research Institute, United States of America

## Abstract

The synthesis of virulence factors by pathogenic bacteria is highly regulated and occurs in response to diverse environmental cues. An array of two component systems (TCSs) serves to link perception of different cues to specific changes in gene expression and/or bacterial behaviour. Those TCSs that regulate functions associated with virulence represent attractive targets for interference in anti-infective strategies for disease control. We have previously identified PA2572 as a putative response regulator required for full virulence of *Pseudomonas aeruginosa*, the opportunistic human pathogen, to *Galleria mellonella* (Wax moth) larvae. Here we have investigated the involvement of candidate sensors for signal transduction involving PA2572. Mutation of *PA2573*, encoding a probable methyl-accepting chemotaxis protein, gave rise to alterations in motility, virulence, and antibiotic resistance, functions which are also controlled by PA2572. Comparative transcriptome profiling of mutants revealed that PA2572 and PA2573 regulate expression of a common set of 49 genes that are involved in a range of biological functions including virulence and antibiotic resistance. Bacterial two-hybrid analysis indicated a REC-dependent interaction between PA2572 and PA2573 proteins. Finally expression of *PA2572* in the *PA2573* mutant background restored virulence to *G. mellonella* towards wild-type levels. The findings indicate a role for the orphan chemotaxis sensor PA2573 in the regulation of virulence and antibiotic tolerance in *P. aeruginosa* and indicate that these effects are exerted in part through signal transduction involving PA2572.

## Introduction

Bacterial genomes encode a multitude of sensory systems that allow them to detect alterations in their extracellular and intracellular environments and to respond appropriately. Two-component signal transduction systems (TCS) comprise a sensor kinase, which in many cases is directly responsible for signal perception, and a response regulator that has a receiver domain and an output domain that exerts a particular regulatory action. Chemosensory two-component systems contain additional machinery for signal transduction such as methyl-accepting chemotaxis proteins (MCPs). Importantly, some TCSs have been shown to play a role in the virulence of diverse pathogenic bacteria including the human pathogen *Pseudomonas aeruginosa* [1]–[3]. As no such systems have been found in mammals, TCSs could provide attractive targets for anti-infective therapies [4]. Such a strategy for *P. aeruginosa* requires identification of those signalling systems that are key for virulence from the inventory of 26 MCPs and 47 other TCSs encoded by the genome [5].

Although most response regulators contain DNA-binding output domains and serve as transcriptional regulators, other classes of output domain are seen [6]. These classes include enzymes involved in the turnover of the nucleotide second messenger cyclic di-GMP [7], [8]; GGDEF domain proteins are diguanylate cyclases (DGCs) catalyzing cyclic di-GMP synthesis whereas EAL and HD-GYP domain proteins are phosphodiesterases (PDEs) catalyzing degradation of cyclic di-GMP. Such regulators can exert their effects via an influence on cyclic di-GMP levels but also through protein-protein interactions [9]–[11]. Of particular interest here are the proteins of *P. aeruginosa* with an HD-GYP domain. The *P. aeruginosa* PAO1 proteome contains three HD-GYP domain proteins. PA4108 and PA4781 are enzymatically active phosphodiesterases, whereas PA2572 encodes a non-canonical variant YN-GYP and does not have detectable activity in cyclic di-GMP degradation. Despite these differences, all three HD-GYP domain proteins play a role in the regulation of biofilm formation, production of virulence factors and virulence in the *Galleria mellonella* model of infection [12]. Both PA4781 and PA2572 have an N-terminal Receiver (REC) domain, which suggests that they are regulatory components of signal transduction pathways associated with virulence. In this study we examined the involvement of candidate sensory proteins in signal transduction involving PA2572.

Genes flanking *PA2572* on the PAO1 chromosome encode candidate sensors for signal transduction involving PA2572; PA2571 is a probable two-component sensor kinase, and PA2573 is a probable methyl-accepting chemotaxis protein (Fig. S1). Although the three genes are predicted to be in separate transcriptional units, *PA2573* is co-expressed with *PA2572* under several conditions including aerobic growth in the presence of nitrate and in bacteria co-cultured with respiratory epithelia cells [13]–[15]. We investigated the potential interplay between these sensors and the regulator PA2572 utilizing bacterial two-hybrid assay to assess protein-protein interactions together with phenotypic and comparative transcriptome profiling analysis of mutants. The findings indicate a role for the orphan chemotaxis sensor PA2573 in the regulation of virulence and antibiotic tolerance in *P. aeruginosa* and suggest that these effects are exerted in part through signal transduction involving PA2572.

**Figure 1:**
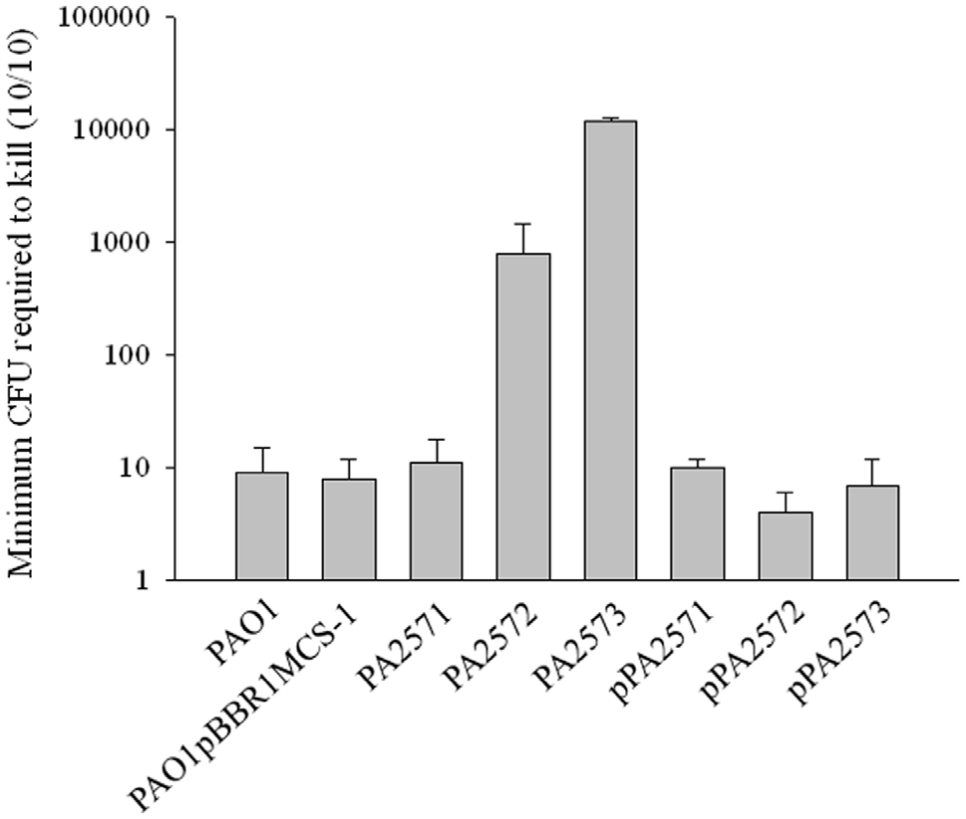
Pathogenesis of strains in the Wax Moth model of infection. Effects of mutation inactivating genes encoding *PA2571*, *PA2572* and *PA2573* on virulence in the *Galleria mellonella* model 24 hours post-infection. Virulence potential of *P. aeruginosa* strains was assessed based on the number of CFUs required to kill a group of ten larvae. Groups injected with PBS and groups without any inoculation served as negative controls. Mutation of *PA2572* and *PA2573* led to reduced virulence compared to wild-type PAO1 or wild-type with the cloning vector pBBR1MCS-1, which were equivalent. Complemented strains (indicated by pPA2572 and pPA2573) had wild-type virulence. Mutation of *PA2571* had no effect. Error bars represent mean ± standard deviation of at least three experiments.

## Materials and Methods

### Bacterial Strains, Plasmids, Primers and Culture Conditions

*Pseudomonas aeruginosa* and *Escherichia coli* strains were cultured in Luria-Bertani (LB) broth at 37°C. For solid media, 1.5% agar was added. Bacterial strains used in this study are listed in Table S1. Antibiotics, obtained from Sigma Chemical Company, gentamycin (Gm), chloramphenicol (Cm) and tetracycline (Tc) were added where appropriate. Oligonucleotide primers were synthesized by MWG (Germany) and are listed in Table S2.

### Construction and Complementation of *PA2571*, *PA2572*, and *PA2573* Mutants

Gene disruption mutants were created using the suicide plasmid pEX18Gm (Table S1). In short, central fragments of each gene were amplified using primers (Table S2) and cloned into pEX18Gm. Restriction enzymes and T4 ligase used for cloning purposes were obtained from Roche Diagnostics and used according to the manufacturer’s instructions. This construct was isolated using the Qiagen QIAprep Spin Miniprep Kit and subsequently introduced into the wild-type *P. aeruginosa* PAO1 strain by electroporation. Integration results in the formation of a stable Gm^R^ mutant and was confirmed by colony PCR using primers detailed in Table S2. Complementation clones were assembled in pBBR1MCS using wild-type gene amplifications from genomic DNA with primers listed in Table S2. The resulting constructs were introduced into the appropriate disruption mutants by electroporation. These strains were selected on LB agar medium containing Gm (20 µg/ml) and Cm (50 µg/ml) and confirmed by colony PCR.

**Figure 2:**
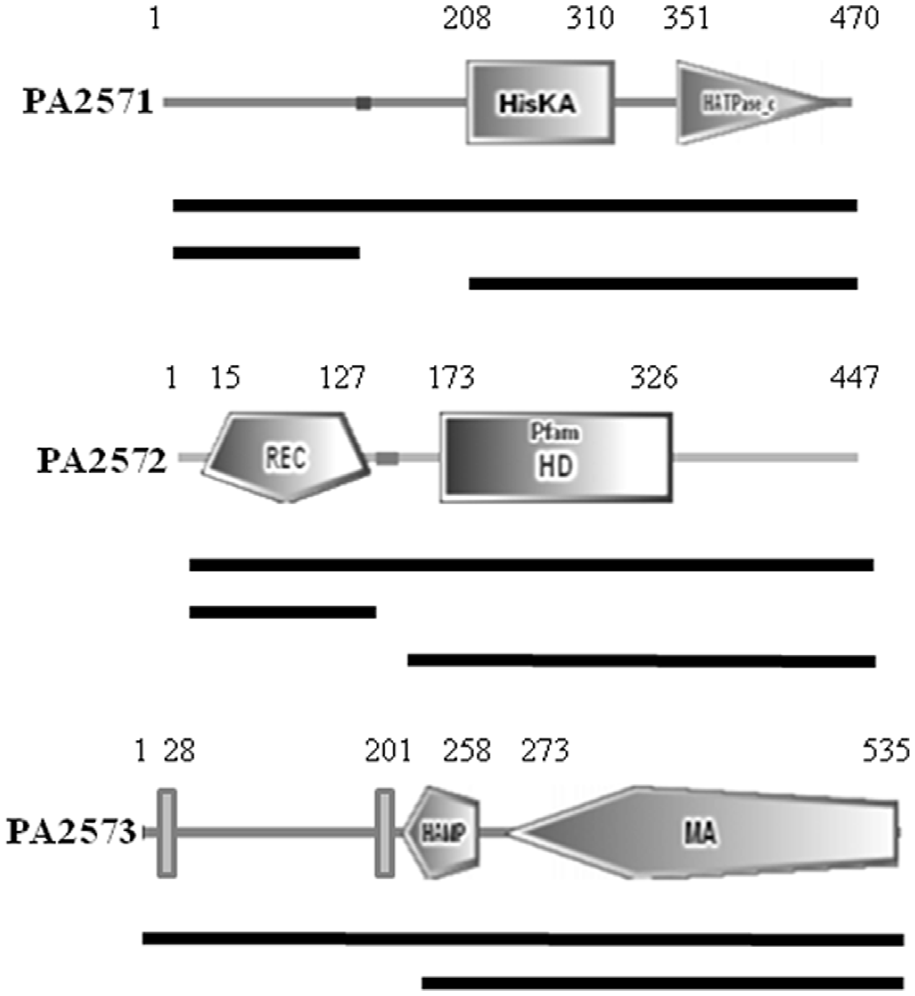
Domain organizations of PA2571, PA2572 and PA2573. Domains were predicted by SMART with amino acid positions indicated. Domain abbreviations are as follows; HisKA (Histidine Kinase A phosphoacceptor domain), HATPase_c (Histidine kinase-like ATPase), REC (Receiver domain), HD (superfamily with predicted or known phosphohydrolase activity), HAMP (Histidine kinase, Adenylyl cyclase, Methyl binding protein, Phosphatase domain), and MA (Methyl-accepting chemotaxis-like domain). Vertical bars represent predicted transmembrane domains (SOUSI). Black lines below figures represent constructs cloned into either pBT or pTRG vectors to assess potential protein-protein interactions using the bacterial two-hybrid assay.

### Motility

Swimming and swarming phenotypes of *P. aeruginosa* strains were assessed on LB media with 0.3% agar and Eiken media with 0.5% agar, respectively. Motility plates were inoculated by sterile tips using freshly streaked cultures grown on LB media. Plates were incubated at 37°C for 24 to 48 hours.

### Pyoverdine and Pyocyanin Production

For pyoverdine production, a conical flask containing 30 ml of F broth, comprised of 20 g/l bacteriological peptone (Oxoid), 1.5 g/l magnesium sulphate hydrated (Fluka), 1.5 g/l dipotassium phosphate (Sigma), and 10 ml/l glycerol, was inoculated with *P. aeruginosa* strains (1∶1000). OD_420_ measurements were taken from supernatants removed from cultures grown to an OD_600_ of 2.0. Pyoverdine production was calculated as OD_420_/OD_600_. For production of pyocyanin, 30 ml of P broth, comprised of 20 g/l bacteriological proteose peptone no. 3 (Oxoid), 1.4 g/l magnesium chloride anhydrous (Sigma), 10 g/l potassium sulphate (Sigma) and 10 ml/l glycerol, was inoculated with *P. aeruginosa* strains (1∶1000). Cultures were grown at 37°C for 24 to 48 hours and 3 ml chloroform was added to supernatant removed from 5 ml of culture. This suspension was vortexed for 10 sec and centrifuged for 5 min at 5,000 rpm. 1.5 ml of chloroform was removed and samples were re-extracted with 2 ml of 0.2N HCl, vortexed for 3 sec and left still to separate. OD_520_ measurements were taken from the top phase and the concentration of pyocyanin determined as described previously [16].

### Antibiotic Susceptibility

Strains were subcultured and grown to logarithmic phase (0.6−0.7 OD_600_) at 37°C, washed twice and resuspended in phosphate buffered saline (PBS) (Sigma). 300 µl of cell suspensions were spread onto 150×20 mm Petri dishes (Sarstedt) containing 60 ml of LB agar and left to dry for 10 min. Antibiotic E-test strips (Biomerieux) were overlaid and plates were incubated at 37°C for 24 hours. Minimum inhibitory concentrations (MIC) (µg/ml) for each antibiotic were recorded in triplicate in two separate experiments for each strain.

### *Galleria mellonella* Virulence Assay

Virulence of *P. aeruginosa* strains was assessed as previously described by Ryan *et al*. [12]. Briefly, bacteria from overnight cultures were washed twice and re-suspended in PBS. Serial 10-fold dilutions were made in PBS and 10 µL aliquots were injected into each larva, with 10 larvae inoculated for each dilution. A minimum of three different dilutions were inoculated per strain in three independent experiments. Uninoculated larvae and larvae injected with PBS alone served as control groups for each experiment. Larvae were incubated in the dark at 37°C in Petri dishes lined with Whatman paper. Minimum inoculum required to kill all 10 larvae was determined for each strain.

### RNA Extraction

Three independent cultures of each *P. aeruginosa* strain was sub-cultured and grown to logarithmic phase (0.6−0.7 OD_600_) at 37°C in LB broth without selection. 800 µl of RNA protect (Qiagen) was added to 400 µl culture and incubated at room temperature for 5 min. Cell suspensions were centrifuged, the supernatant was disregarded, and pellets were stored at −80°C. After thawing, 100 µl TE-lysozyme (400 µg/ml) was added and samples were incubated at room temperature. Total RNA was isolated using the RNeasy Mini Kit (Qiagen) whereby cells were homogenized utilizing a 20-gauge needle and syringe. Samples were treated with DNase (Ambion) according to manufacturer’s instructions and the removal of DNA contamination was confirmed by PCR.

### Transcriptome Profiling and Analysis

RNA samples were analysed using the Affymetrix GeneChip technology platform at University College Dublin Conway Institute of Biomolecular and Biomedical Research. Analysis of microarray data was achieved using GeneSpring Multi-Omic Analysis Version 11.5 (Agilent Technologies). Two-fold changes in gene expression between mutant and the wild-type parental *P. aeruginosa* PAO1 strain were considered to be significant (p≤0.05). The microarray data set has been deposited into ArrayExpress Database under the accession number [pending].

### Semi-quantitative and Quantitative Real-time PCR

Semi-quantitative and quantitative RT-PCRs were used to validate microarray data. Reverse transcription PCR was achieved using a cDNA synthesis kit (Promega) according to the manufacturer’s instructions. Specific RT-PCR primers (Table S2) were used to amplify central fragments of approximately 200 bp in length from different genes. Semi-quantitative RT-PCRs were completed using 250 ng/µl cDNA template and PCR Mastermix (Promega) for 24–36 cycles. For qRT-PCRs, quantification of gene expression and melting curve analysis were completed using a LightCycler (Roche) and Platinum SYBR Green qPCR Supermix-UGD (Invitrogen) was used according to manufacturer’s instructions. The constitutively expressed housing keeping gene, *16S rRNA* was used as a reference to standardize all samples and replicates.

### Bacterial Two-hybrid Analysis

BacterioMatch II Two-Hybrid System Vector Kit (Agilent Technologies) was used to evaluate protein-protein interactions of *P. aeruginosa* PAO1 proteins in *E. coli*. This analysis was carried out according to manufacturer’s instructions. The pBT bait plasmid and the pTRG target plasmid containing partial and full-length gene fragments were constructed using primers listed in Table S2. A co-transformant containing empty pBT and pTRG vectors served as a negative control and a co-transformant containing pBT-LGF2 and pTRG-GAL11P served as a positive control, validated by growth of colonies on M9+ His-dropout media containing 5 mM 3-amino-1,2,4, triazole (3-AT). Co-transformations for detecting interactions between bait and target proteins were plated on selective screening medium (SSM) (5 mM 3-AT) and on non-selective screening medium (NSSM) (without 3-AT) to serve as a control.

**Table 1:** Bacterial two-hybrid analysis investigating potential protein-protein interactions.

Target Protein (pTRG vector)						
Bait Protein (pBT vector)	PA2571 RECdomain	PA2571 HKdomain	PA2571 Full length	PA2573 - TMdomain	PA2573 Fulllength	PA4781 REC domain
PA2572 REC domain	−	−	−	+	+	ND
PA2572 YN-GYP domain	−	−	−	−	−	ND
PA2572 Full length	−	−	−	+	+	ND
PA2573 - TM domain	ND	ND	ND	ND	ND	−

(+) indicates a positive protein-protein interaction based on growth of colonies on Selective Screening Media (SSM) (M9+ His-dropout media containing 5 mM 3-AT). (−) indicates a negative interaction based on lack of growth on SSM. (-TM) represents a PA2573 construct without the N-terminal transmembrane domains. Results were observations from three independent experiments.

### Expression of Individual Domains and Full Length PA2572 in *PA2572* and *PA2573* Mutant Backgrounds

PA2572 complementation clones were created using pME6032 plasmid (Table S1). Briefly, primers (Table S2) were used to amplify DNA encoding the REC domain, the YN-GYP domain and the full length *PA2572* gene. The amplicons were sequenced and were cloned into pME6032. These three constructs were transformed into Top 10 *E. coli* cells (Invitrogen) and isolated using the Qiagen QIAprep Spin Miniprep Kit. Constructs were subsequently introduced into the PA2572 and PA2573 mutant strains and into the parental PAO1 strain by electroporation. As an additional control, the empty pME6032 vector was introduced into all strains. The presence of pME6032 containing the relevant DNA fragment was confirmed using primers (Table S2) corresponding to the multiple cloning site of the plasmid.

## Results

### Mutation of *PA2572* and *PA2573* but not *PA2571* Influences Virulence of *P. aeruginosa* in the Greater Wax Moth Larvae Model

Virulence of *P. aeruginosa* strains in the *G. mellonella* larvae model was assessed by determination of the minimum CFU required to kill all of a group of 10 larvae in which each member received an inoculum from a 10-fold serial dilution series (see Materials and Methods). This minimum inoculum for the parental PAO1 strain was 9±6 CFU (*n* = 8) (Fig. 1). The minimum inoculum for the *PA2571* mutant was similar to that of the wild-type, suggesting no effect on virulence (Fig. 1). In contrast, considerably higher numbers of the *PA2572* and *PA2573* mutant strains were required, indicating that both PA2572 and PA2573 contribute to the full virulence of *P. aeruginosa* in the *G. mellonella* model of infection. These findings for PA2572 confirm our earlier published work [12]. Importantly, complementation of the *PA2572* and *PA2573* mutants with wild-type copies of the appropriate genes restored virulence to wild-type levels (Fig. 1).

**Figure 3:**
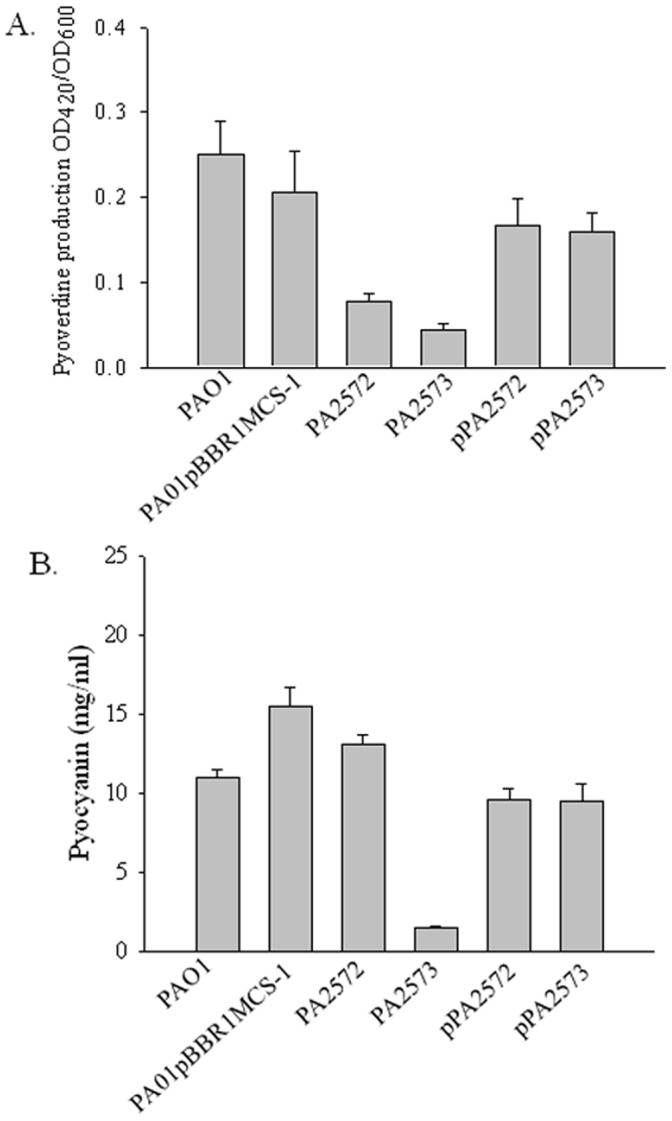
Production of virulence factors. Effects of mutations of *PA2572* and *PA2573* on production of pyoverdine (A) and pyocyanin (B) in *P. aeruginosa*. Complemented strains (indicated by pPA2572 and pPA2573) had wild-type levels of the factors. Phenotypic effects of mutations were complemented with reintroduction of genes into mutant strains. Error bars represent the mean ± standard deviation of three independent experiments in triplicate.

### Bacterial Two-hybrid Analysis Reveals Direct Interaction between PA2572 and PA2573

The BacterioMatch II Two-Hybrid System was used to evaluate potential protein-protein interactions between the putative regulator PA2572 and the predicted sensor proteins PA2571 and PA2573. For these experiments, the pBT bait plasmid and the pTRG target plasmid expressing full-length PA2571, PA2572, and PA2573 or different domains of the proteins were constructed following PCR amplification of the appropriate DNA fragments using primers listed in Table S2. The domain structures of the full-length proteins and protein fragments used in this analysis are illustrated in Fig. 2. Detection of interactions between bait and target proteins was based on growth of co-transformant colonies on SSM media containing 5 mM 3-AT (Table 1).

**Figure 4:**
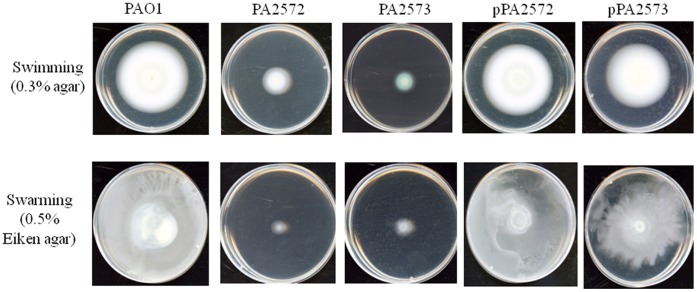
Motility of strains. Motility phenotypes of *P. aeruginosa* for swimming (0.3% agar) and swarming (0.5% Eiken agar) after 24 hours at 37°C. Mutant strains (indicated by PA2572 and PA2573) have reduced swimming and swarming, whereas complemented strains (indicated by pPA2572 and pPA2573) had wild-type levels of motility. Each plate is representative of the motility phenotype observed for strains in triplicate in three independent experiments.

No interaction was detected between PA2572 and PA2571 in experiments using either the full-length proteins or any of the domains of these proteins. In contrast, protein-protein interactions between PA2572 and the predicted methyl-accepting chemotaxis protein PA2573 were detected in three independent experiments. Furthermore, interactions between PA2572 and the predicted cytoplasmic portion of PA2573 (comprising the HAMP and methyl-accepting domains) and between the REC domain of PA2572 and full-length PA2573 were detected (Fig. 2). No protein-protein interaction was detected between PA2573 and the REC domain of PA4781, an enzymatically-active HD-GYP domain protein, suggesting a specificity of interaction with PA2572. Taken together, these results suggest an interaction between PA2572 and PA2573 mediated by the REC domain of PA2572 and the cytoplasmic portion of PA2573, but no interaction between PA2572 and PA2571. These findings and the virulence phenotypes of different mutants outlined above suggest no role for PA2571 in virulence-related signal transduction involving PA2572. As a consequence, subsequent experiments focussed on the role of PA2573.

### Comparison of Phenotypic Effects of Mutation of *PA2572* and *PA2573*

The *PA2572* and *PA2573* mutant strains were assessed for phenotypes associated with *P. aeruginosa* virulence including production of pyocyanin and the siderophore pyoverdine as well as motility. Mutation of *PA2572* and *PA2573* resulted in strains with a reduced ability to produce pyoverdine (Fig. 3A). Complementation of the mutations with wild-type copies of the genes cloned into the pBBR1MCS vector restored pyoverdine levels to near wild-type. Mutation of *PA2573* also led to a reduction of pyocyanin production, although this effect was not seen in the *PA2572* mutant (Fig. 3B). Complementation of the PA2573 mutations restored pyocyanin levels to wild-type (Fig. 3B). Disruption of *PA2572* and *PA2573* had a notable influence on both swimming and swarming motility; mutant strains displayed a significant reduction in motility compared to PAO1 (Fig. 4). Complementation of mutant strains restored both swarming and swimming motility to wild-type levels.

**Figure 5:**
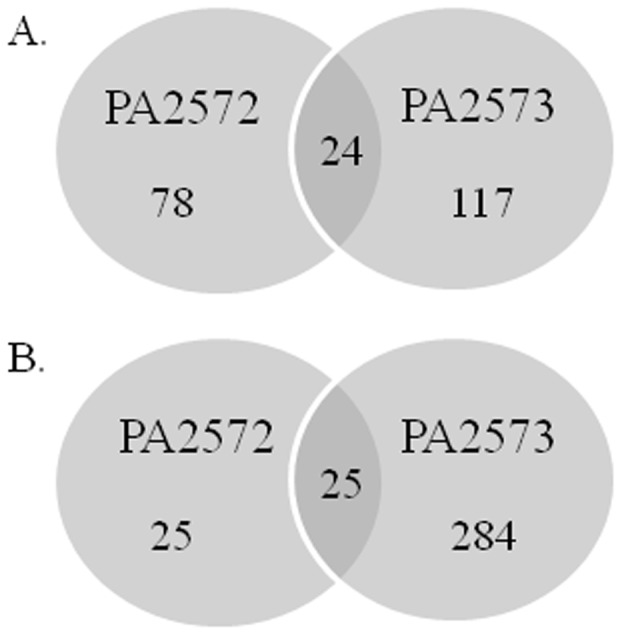
Venn diagrams representing unique and overlapping gene regulation in PA2572 and PA2573 mutants. Mutant strains were compared to the parental PAO1 strain during exponential growth phase (0.6–0.7 OD_600_) in LB media. Genes were considered to have significant alteration in expression based on two-fold changes compared with wild-type. The numbers of genes regulated in an upward direction (A) and a downward direction (B) are shown.

### Comparative Global Gene Expression Profiles in *PA2572* and *PA2573* Mutant Strains

To compare the influence of PA2572 and PA2573 on gene expression, the transcriptomes of *PA2572* and *PA2573* mutant strains and wild-type were compared through microarray analysis using the *P. aeruginosa* Affymetrix GeneChip. For these experiments, RNA was isolated from bacterial strains growing in exponential phase in LB medium. Transcriptome profile analysis revealed that expression of 152 genes was significantly altered (≥2.0-fold) by the disruption of *PA2572* (Fig. 5). Disruption of *PA2573* appeared to have a broader influence on global gene expression as 450 genes were significantly differentially expressed in the mutant compared to PAO1. PA2572 and PA2573 influenced expression of a common set of 49 genes: 24 genes with an increase in expression and 25 genes with a decrease in expression (Fig. 5). These genes were involved in a range of biological functions including virulence, membrane transport, multidrug resistance, amino acid biosynthesis and signal transduction. Additionally, microarray analysis revealed 12 genes whose expression levels were altered in opposite directions following mutation of *PA2572* and *PA2573*. These genes included *pqsA* involved in the biosynthesis of the *Pseudomonas* quinolone signal (PQS) [17] and several genes of the lipopolysaccharide modification operon *arnBCADTEF* [18], [19]. Differentially expressed genes in mutant strains compared to PAO1 with a fold change ≥3.0 are listed in Tables S3 and Table S4 along with their assigned annotations.

**Table 2:** Validation of transcriptome data using quantitative and semi-quantitative real-time PCR.

		Transcriptome	qRT-PCR	Transcriptome	qRT-PCR
Gene	Description	Fold changePA2572/PAO1 (>2.0)	Fold changePA2572/PAO1	Fold changePA2573/PAO1(>2.0)	Fold changePA2573/PAO1
*PA1425*	probable ATP-binding component of ABCtransporter	4.51	2.89	NS	−0.49
*PA2019*	*mexX*, RND multidrug efflux membranefusion protein precursor	16.92	36.5	12.66	12.8
*PA3530*	conserved hypothetical protein	5.09	9.03	4.66	2.31
*PA4290*	probable chemotaxis transducer	2.86	5.72	NS	1.65
*PA4307*	*pctC*, chemotactic transducer PctC	−2.20	0.95	−3.73	−9.06
*PA4527*	*pilC*, still frameshift type 4 fimbrialbiogenesis protein PilC	NS	0.62	−4.83	−8.12
*PA4825*	*mgtA*, Mg(2+) transport ATPase, P-type 2	10.89	14.1	NS	−0.23
		**Transcriptome**	**Semi-q RT-PCR**	**Transcriptome**	**Semi-q RT-PCR**
**Gene**	**Description**	**Fold change** **PA2572/PAO1 (>2.0)**	**Fold change** **PA2572/PAO1**	**Fold change** **PA2573/PAO1(>2.0)**	**Fold change** **PA2573/PAO1**
*PA0517*	*nirC*, probable c-type cytochrome precursor	NS	NC	−14.71	−
*PA0830*	hypothetical protein	−4.12	–	−3.77	−
*PA1326*	*ilvA2*, threonine dehydratase	11.80	+	5.57	+
*PA1710*	*exsC*, ExsC, exoenzyme S synthesis proteinC precursor	NS	NC	−4.35	−
*PA1718*	*pscE*, type III export protein PscE	NS	NC	−30.95	−
*PA1797*	hypothetical protein	56.20	+	NS	NC
*PA2513*	*antB*, anthranilate dioxygenase small subunit	NS	NC	29.35	+
*PA3190*	probable binding protein component(ABC sugar transporter)	−6.65	−	NS	NC
*PA4825*	*mgtA*, Mg(2+) transport ATPase	10.89	+	NS	NC
*PA5471*	hypothetical protein	7.01	+	3.14	+

Abbreviations and symbols: NS (Not significant, fold change was less than 2.0), RND (Resistance-Nodulation-Cell Division), + (represents increased gene expression in mutant strain based on increased abundance of PCR amplicon compared to PAO1), − (represents decreased gene expression in mutant strain based on decreased abundance of PCR amplicon compared to PAO1), NC (no apparent change in abundance was observed).

Notably, expression of several genes encoding components of the *P. aeruginosa* type III secretion system were down-regulated in both *PA2572* and *PA2573* mutants compared to the wild-type. These virulence genes included those encoding PopB and PopD, which are essential for the translocation process, the chaperone PcrH, and the secreted toxin ExoS [20], [21]. Disruption of *PA2572* and *PA2573* also led to common expression of a number of genes involved in signal transduction including *PA4781* encoding an HD-GYP domain phosphodiesterase [12], *PA3271* encoding a probable two-component sensor, and *pctC* encoding a chemotactic transducer.

Both semi-quantitative and qRT-PCR methods were used to confirm alterations in gene expression revealed by microarray analysis (Table 2). The genes selected for these analyses represented those with a range of fold change of expression and of diverse functional classes. The relative expression levels of seven genes measured using qRT-PCR and ten genes using semi-quantitative RT-PCR reflected in each case the differences in gene expression observed by transcriptome analysis (Table 2).

**Table 3:** Transcriptome profiles highlight regulation of genes related to antibiotic resistance in PA2572 and PA2573 mutants.

		Transcriptome	Transcriptome		
Gene	Description	Fold change PA2572/PAO1 (>2.0)	Fold change PA2573/PAO1 (>2.0)	Antibiotic*	Ref.
*PA0958*	*oprD*, Basic amino acid, basic peptide andimipenem outer membrane porin OprD precursor	−3.24	−2.01	Meropenem	[33], [34]
*PA1797*	hypothetical protein harboring beta-lactamaseconserved domain (Pfam)	56.20	NS	β-lactam (Ceftazidime)/Polymyxin B	[19]
*PA2018*	*mexY*, Resistance-Nodulation-Cell Division (RND)multidrug efflux transporter	11.14	9.04	Amikacin/Tobramycin	[22], [23], [35]
*PA2019*	*mexX*, Resistance-Nodulation-Cell Division (RND) multidrugefflux membrane fusion protein precursor	16.92	12.66	Amikacin/Tobramycin	[22], [23], [35]
*PA4200*	hypothetical protein harboring metallo Beta-lactamase_Band B_2 domains (Pfam)	−3.82	NS	β-lactam (Ceftazidime)	
*PA4599*	*mexC*, Resistance-Nodulation-Cell Division (RND) multidrug efflux membrane fusion protein MexC precursor	4.45	NS	Ciprofloxacin	[35], [36]
*PA4776*	*pmrA*, two-component regulator system responseregulator PmrA	3.60	−2.01	Polymyxin B/Colistin	[18], [37]
*PA4777*	*pmrB*, two-component regulator system signalsensor kinase PmrB	4.36	NS	Polymyxin B/Colistin	[18], [37]

* Antibiotics in which the expression of listed genes has been implemented in resistance or susceptibility. References are indicated.

### PA2572 and PA2573 Regulate Expression of Genes Involved in the Antibiotic Tolerance of *P. aeruginosa*

Transcriptome profiling revealed that both PA2572 and PA2573 regulate a number of genes associated with antibiotic tolerance (Table 3). Significant increases in expression (∼9- to 17-fold) were observed for *mexX* and *mexY* that encode Resistance-Nodulation-Cell Division (RND) multidrug efflux proteins involved in resistance to aminoglycoside antibiotics in *P. aeruginosa* [22], [23]. The *oprD* gene, that encodes an outer membrane porin that facilitates the uptake of carbapenem antibiotics [24], was also seen to be downregulated in both mutant strains compared to the wild-type. Conversely, disruption of *PA2572* and *PA2573* resulted in the differential regulation of *pmrA,* a gene encoding a two-component regulator associated with resistance to cationic antimicrobial peptides [18]; expression of *pmrA* increased in the *PA2572* mutant but decreased in the *PA2573* mutant when compared to wild-type. Additionally, mutation of *PA2572* also led to the up-regulation of *pmrB* encoding the sensor kinase component of this two-component system.

These effects on gene expression prompted us to examine the antibiotic tolerance of the different *P. aeruginosa* strains. Minimum inhibitory concentrations (MIC) of antibiotics towards *P. aeruginosa* strains grown to exponential phase were assessed using E-test strips (Biomerieux) after 24 hours (Table 4). Both *PA2572* and *PA2573* mutants demonstrated substantially increased tolerance to the aminoglycosides amikacin and tobramycin compared to the parental PAO1 strain (Table 4). Complementation of the mutations restored susceptibility to both aminoglycosides towards wild-type levels. Mutation of *PA2572* led to increased tolerance to the cationic antimicrobial peptides, polymyxin B and colistin, although mutation of *PA2573* had the opposite effect. Intriguingly, genes involved in resistance appeared to be divergently transcribed. Tolerance to ceftazidime, meropenem and ciprofloxacin was minimally affected by disruption of *PA2572* and *PA2573*.

**Table 4:** Minimum inhibitory concentrations of antibiotics toward *P. aeruginosa* strains grown on LB agar.

MIC (µg/ml)							
Strain	Amikacin	Tobramycin	Polymixin B	Colistin	Ceftazidime	Ciprofloxacin	Meropenem
PAO1	6.67	1.41	2.83	4.17	4.33	0.21	0.25
PA2572	>254	32	4.67	10	2.33	0.21	0.29
PA2573	122.67	14	1.83	1.67	3.83	0.25	0.38
pPA2572	11.33	1.83	4	7.33	4.33	0.21	0.46
pPA2573	10	2	2.67	4	4	0.29	0.38

MICs were measured using Antibiotic E-test strips (Biomerieux) and results shown are the average of 6 independent experiments.

### Expression of the PA2572 YN-GYP Domain Partially Restores Virulence of the *PA2573* Mutant

**Figure 6:**
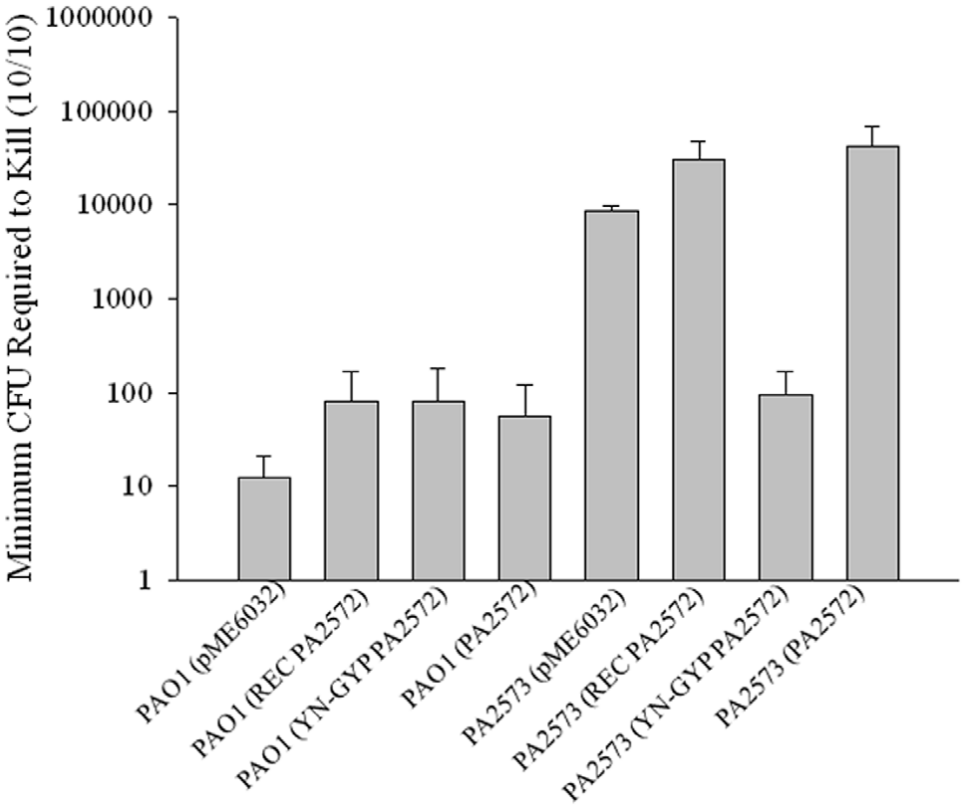
Pathogenesis of strains expressing PA2572. Effects of expressing partial and full-length fragments of PA2572 in the PA2573 mutant and in the wild-type on virulence in the *Galleria mellonella* model. Expression of the empty pME6032 vector in strains served as a control. Virulence potential of *P. aeruginosa* strains was assessed based on the number of CFU required to kill a group of ten larvae 24 hours post-infection. Error bars represent mean ± standard deviation of at least three experiments.

The findings described above indicate a role for the PA2573 sensor in the regulation of virulence and antibiotic tolerance in *P. aeruginosa*. The overlap in regulatory influence of PA2572 and PA2573 at the level of gene transcription and on various phenotypes including virulence suggests that these effects may be exerted in part through signal transduction involving PA2572. For a number of two-component systems, it has been shown that over-expression of the response regulator can restore the phenotypes of a mutant lacking the related sensor to wild-type phenotypes [25], [26]. Accordingly, we assessed the effects of expression of the full length PA2572, and the component REC or YN-GYP domains on the virulence of the *PA2573* mutant. For this experiment the DNA fragments were cloned into pME6032 and introduced into *PA2573* mutant and wild-type, as a control. Subsequently, all strains were assessed for their virulence potential in the *Galleria mellonella* model of infection (Fig. 6, S2). The empty pME6032 vector had no effect on the virulence of *PA2573* mutant or wild-type strain (Fig. 6). While expression of the REC domain and the full length PA2572 in the *PA2573* mutant had a small negative influence on virulence, introduction of the YN-GYP domain resulted in a significant increase in virulence (Fig. 6).

## Discussion

The work in this paper had the aim of identifying signalling partners for the putative response regulator PA2572, which we have previously shown to contribute to the virulence of *P. aeruginosa* to *Galleria mellonella* larvae. Two candidates were examined: the probable two-component sensor kinase PA2571 and probable methyl-accepting chemotaxis protein PA2573. It has been shown previously that *PA2573* is co-expressed with *PA2572* under several conditions including aerobic growth in the presence of nitrate and in bacteria co-cultured with respiratory epithelia cells [13]–[15]. Here we present a number of lines of evidence that suggest regulatory interplay between PA2573 and PA2572. We have shown that PA2572 and PA2573 are both involved in the regulation of motility, virulence and antibiotic tolerance and influence the expression of a common set of 49 genes. Furthermore, we have demonstrated using bacterial two-hybrid analysis that protein-protein interactions can occur between PA2573 and PA2572 and that these involve the REC domain of PA2572. In contrast, PA2571 does not influence virulence and no interaction with PA2572 was evident in bacterial two-hybrid analysis. The findings indicate for the first time a role for the orphan chemotaxis sensor PA2573 in the regulation of virulence and antibiotic tolerance in *P. aeruginosa* and suggest that the action of PA2573 is exerted in part through signal transduction involving PA2572.

For a number of two-component systems it has been shown that over-expression of the response regulator can restore to wild-type the phenotypes of a mutant lacking the related sensor. Overproduction of response regulators is often thought to mimic the physiological phosphorylation response. For many two-component regulators, interactions between the unphosphorylated receiver domain and the effector domain prevent effector domain activity by restricting it in an unfavorable conformation; phosphorylation of the REC domain relieves this inhibition [27]. However over-expression of PA2572 had a small but negative effect on the virulence of the *PA2573* mutant. Intriguingly, over-expression of the YN-GYP domain of PA2572 gave a substantial increase in virulence, which was restored towards wild-type levels. We speculate that this effect is due to release of the YN-GYP effector domain from a negative influence of the REC domain.

While genes encoding cognate two-component signal transduction proteins are often found flanking one another on the genome, there is evidence that a number of signalling systems employ orphan histidine kinases (HK) and response regulators not encoded in the genomic vicinity of their partners [28]–[32]. Under the conditions tested, PA2571 does not appear to have a role in virulence of *P. aeruginosa* and there was no evidence for an interaction between PA2571 and PA2572. While it is possible that an orphan HK is part of the machinery for this proposed signalling pathway involving PA27573 and PA2572, we cannot exclude that that regulatory interplay between PA2571 and PA2572 occurs under different environmental conditions.

Mutation of *PA2573* influenced the expression of a broader range of genes than mutation of *PA2572*, suggesting that PA2573 may interact with additional response regulators in other signaling pathways. However mutation of either *PA2573* or *PA2572* leads to enhanced expression of genes associated with antibiotic tolerance, which is reflected in increased MICs for the aminoglycoside antibiotics amikacin and tobramycin in these mutant strains. Mutation of *PA2573* or *PA2572* both lead to the down-regulation of several genes encoding virulence factors including those involved in type III secretion (*popB*, *popD*, *pcrH*, as well as the secreted effector *exoS* (Tables S3 and S4). This is reflected in a decreased virulence of both mutants.

Several laboratories have described modified chemotaxis systems that are linked to alterations in the levels of nucleotide second messengers in *P. aeruginosa.* The Wsp system functions to regulate cellular processes such as EPS production and biofilm formation [7], [8]. Transduction of environmental signals through the MCP WspA and a soluble sensor kinase WspE leads to phosphorylation of the response regulator WspR, activating this GGDEF domain protein for the synthesis of cyclic di-GMP. The Chp chemosensory system is linked to cAMP-dependent virulence response in *P. aeruginosa* through modulation of adenylate kinase activity [2]. In a similar fashion, we propose that a novel signalling pathway that includes an orphan chemosensory protein component (PA2573) and a response regulator (PA2572) with a variant HD-GYP domain acts in regulation of factors promoting the virulence and antibiotic tolerance of *P. aeruginosa*. Although the mechanisms by which PA2572 exerts a regulatory action remain obscure, the discovery of a role of PA2573 in virulence in model organisms suggests that further studies of this system are warranted.

## Supporting Information

Figure S1 **Organization of** ***PA2572*** **and flanking genes in the** ***P. aeruginosa*** **PAO1 chromosome.** Region is drawn approximately to scale and arrows represent gene orientation.
(TIF)

Figure S2 **Pathogenesis of the PA2572 mutant expressing PA2572.** Effects of expressing partial and full-length fragments of PA2572 in the PA2572 mutant on virulence in the *Galleria mellonella* model. Expression of the empty pME6032 vector served as a control. Virulence potential of *P. aeruginosa* strains was assessed based on the number of CFU required to kill a group of ten larvae 24 hours post-infection. Error bars represent mean ± standard deviation of at least three experiments.
(TIF)

Table S1 Bacterial strains and plasmids used in this study.
(DOCX)

Table S2 Primers used in this study.
(DOCX)

Table S3 Genes regulated in the PA2572 mutant compared to the wild-type PAO1strain during exponential growth in LB media (>3.0-fold).
(DOCX)

Table S4 Genes regulated in the PA2573 mutant compared to the wild-type PAO1strain during exponential growth in LB media (>3.0-fold).
(DOCX)
