# Supplementary material for: An Orphan Chemotaxis Sensor Regulates Virulence and Antibiotic Tolerance in the Human Pathogen Pseudomonas aeruginosa
Source: PLoS One. 2012 Aug 1;7(8):e42205. doi: 10.1371/journal.pone.0042205 (PMC3411652; doi:10.1371/journal.pone.0042205)
Supplement: Table S1 — Bacterial strains and plasmids used in this study. (DOCX) [file pone.0042205.s003.docx]

**Table S1.** Bacterial strains and plasmids used in this study.

| Strain | Description | Reference |
| --- | --- | --- |
| *Pseudomonas aeruginosa* |  |  |
| PA01 | Wild type | ([1](#_ENREF_1)) |
| PA2571 | PA01 derivative, *PA2571*::Gm^r^ disruption mutant using pEX18Gm | This work |
| PA2572 | PA01 derivative, *PA2572*::Gm^r^ disruption mutant using pEX18Gm | ([2](#_ENREF_2)) |
| PA2573 | PA01 derivative, *PA2573*::Gm^r^ disruption mutant using pEX18Gm | This work |
| pPA2571 | Complemented PA2571 mutant using pBBR1MCS | This work |
| pPA2572 | Complemented PA2572 mutant using pBBR1MCS | ([2](#_ENREF_2)) |
| pPA2573 | Complemented PA2573 mutant using pBBR1MCS | This work |
| PA01 (PME6032) | PA01 derivative, harbouring an empty PME6032 vector | This work |
| PA01(Rec-PA2572) | PA01 derivative, complemented with the Receiver domain of | This work |
|  | *PA2572* using pME6032 |  |
| PA01(YN-GYP-PA2572) | PA01 derivative, complemented with the YN-GYP domain of | This work |
|  | *PA2572* using pME6032 |  |
| PA01(PA2572) | PA01 derivative, complemented with *PA2572* using pME6032 | This work |
| PA2572 (PME6032) | PA2572 mutant harbouring an empty pME6032 vector | This work |
| PA2572(Rec-PA2572) | PA2572 mutant complemented with the Receiver domain of | This work |
|  | *PA2572* using pME6032 |  |
| PA2572(YN-GYP-PA2572) | PA2572 mutant complemented with the YN-GYP domain of | This work |
|  | *PA2572* using pME6032 |  |
| PA2572(PA2572) | PA2572 mutant complemented with *PA2572* using pME6032 | This work |
| PA2573 (PME6032) | PA2573 mutant harbouring an empty pME6032 vector | This work |
| PA2573(Rec-PA2572) | PA2573 mutant complemented with the Receiver domain of | This work |
|  | *PA2572* using pME6032 |  |
| PA2573(YN-GYP-PA2572) | PA2573 mutant complemented with the YN-GYP domain of | This work |
|  | *PA2572* using pME6032 |  |
| PA2573(PA2572) | PA2573 mutant complemented with *PA2572* using pME6032 | This work |
|  |  |  |
| *Escherichia coli* |  |  |
| Top 10 | Chemically competent intermediate host, plasmid free | Invitrogen |
|  |  |  |
| Plasmids |  |  |
| pEX18Gm | Broad-host-range allelic exchange vector, Gm^r^ | ([3](#_ENREF_3)) |
| pBBR1MCS | Broad-host-range cloning vector, Cm^r^ | ([4](#_ENREF_4)) |
| pME6032 | Shuttle vector, Tc^r^ | ([5](#_ENREF_5)) |

**References**

1. Holloway BW, Krishnapillai V, & Morgan AF (1979) Chromosomal genetics of Pseudomonas. *Microbiological reviews* 43(1):73-102.

2. Ryan RP*, et al.* (2009) HD-GYP domain proteins regulate biofilm formation and virulence in Pseudomonas aeruginosa. *Environmental microbiology* 11(5):1126-1136.

3. Hoang TT, Karkhoff-Schweizer RR, Kutchma AJ, & Schweizer HP (1998) A broad-host-range Flp-FRT recombination system for site-specific excision of chromosomally-located DNA sequences: application for isolation of unmarked Pseudomonas aeruginosa mutants. *Gene* 212(1):77-86.

4. Kovach ME*, et al.* (1995) Four new derivatives of the broad-host-range cloning vector pBBR1MCS, carrying different antibiotic-resistance cassettes. *Gene* 166(1):175-176.

5. Heeb S, Blumer C, & Haas D (2002) Regulatory RNA as mediator in GacA/RsmA-dependent global control of exoproduct formation in Pseudomonas fluorescens CHA0. *Journal of bacteriology* 184(4):1046-1056.
