# Supplementary material for: An Orphan Chemotaxis Sensor Regulates Virulence and Antibiotic Tolerance in the Human Pathogen Pseudomonas aeruginosa
Source: PLoS One. 2012 Aug 1;7(8):e42205. doi: 10.1371/journal.pone.0042205 (PMC3411652; doi:10.1371/journal.pone.0042205)
Supplement: Table S2 — Primers used in this study. (DOCX) [file pone.0042205.s004.docx]

**Table S2.** Primers used in this study.

| Primer | Sequence |
| --- | --- |
| Strain construction |  |
| PA2571 F | CGCCAAGTGGAACGTGACG |
| PA2571 R | GCAGATCGGTCCACTGCC |
| pPA2571 F | AGCAGCGGATCCATGCACGCGGCGGCTGGG |
| pPA2571 R | CCCGGGGATATCTTAATTTCCGTGGGCTCCGCC |
| PA2573 F | GCTCGCGGGCGGCATC |
| PA2573 R | GGACTGGTTGACCACGTCG |
| pPA2573 F | AGCAGCGGATCCATGAACATTCGGCAGAGAATCC |
| pPA2573 R | CCCGGGGATATCTCAGATCTTGAAGCGCTCCAC |
|  |  |
| RT-PCR |  |
| 16s F | cggtccagactcctacggg |
| 16s R | ttaccgcggctgctggcac |
| PA0517 F | cgttcgcctgtcctcttccgg |
| PA0517 R | ggcccaggggggcatcggggt |
| PA0830 F | atcgactccgtgcgccactac |
| PA0830 R | gcggtgaggtgctccagggccg |
| PA1326 F | actcccgaactcaaggtcaag |
| PA1326 R | tcccggcgatcaggctgccgc |
| PA1425 F | GCGCATTCCTCGACGACGCCG |
| PA1425 R | CCGAGGGTCGACAGCTCGACG |
| PA1710 F | ctcgacgaggagggcatggcg |
| PA1710 R | ggtcagttgcgctgcgagaat |
| PA1718 F | acagccttggaaacgcgactg |
| PA1718 R | agtcctctgtgagctgcgaaa |
| PA1797 F | cgcttccatgccgaccagagc |
| PA1797 R | gcaaagggtccagcaggtgct |
| PA2018 F | cagcgccacggtgaaggtcga |
| PA2018 R | cacgccctccgggaagtagcg |
| PA2019 F | gcaccgtgctgttccagatcg |
| PA2019 R | gcgggtcagcggtgtcggcga |
| PA2513 F | ggacggctacctcgcgcagtt |
| PA2513 R | cagttgacccgcactcgcagc |
| PA3190 F | ggcaagtgggacagcctgctg |
| PA3190 R | agcaccaggttctcgaacacg |
| PA3530 F | TACGTCTGCCTCTGCCAAGGT |
| PA3530 R | GCCGTAGTCCCGAACGCCGGT |
| PA4290 F | GGAAAGCCAGATTACCGAGAT |
| PA4290 R | AGGCTGACCTCGGCGAGGCGC |
| PA4307 F | GCGGCACCTTCACCATGCGCC |
| PA4307 R | CGGCGACGCCCAGCAGTTGAC |
| PA4527 F | CAACTCACTGAGAAAGAAACC |
| PA4527 R | ACGATCAAGGCAACGATGATG |
| PA4825 F | ggcgtcatcatcgtcctctgc |
| PA4825 R | cgcgattcgatcaggcggatg |
| PA5471 F | cgacatcggctgtggcatggc |
| PA5471 R | cagttgcaggcgatcggcatc |
|  |  |
| Bacterial Two-Hybrid |  |
| PA2571 F (Rec) (FL) | AGCAGCGAATTCCCATGAACATTCGGCAGAGAATCC |
| PA2571 R (Rec) | CCCGGGCTCGAGCGCGCCTACCGACGGCATC |
| PA2571 F2 (HisK) | AGCAGCGAATTCC(C)GGATATTCCCGTTCTAGAGC |
| PA2571 R (HisK) (FL) | CCCGGGCTCGAGTTAATTTCCGTGGGCTCC |
| PA2573 F (FL) | AGCAGCGAATTCCCATGAACATTCGGCAGAGAATCC |
| PA2573 F2 (-TM) | AGCAGCGAATTCCCCTGCGCGCCGTCCTGCAGCC |
| PA2573 R (FL) | CCCGGGCTCGAGTCAGATCTTGAAGCGCTCCAC |
| PA2572 F (Rec) (FL) | AGCAGCGAATTCCATGAACGATAGCGCACCTCC |
| PA2572 R (Rec) | CCCGGGCTCGAGCGACTGGCGCAGGGTCAGC |
| PA2572 F (YN-GYP) | AGCAGCGAATTCCCTCGAATACCTGCATTCCG |
| PA2572 R (YN-GYP)(FL) | CCCGGGCTCGAGCTAGGTCGTCGACTCCG |
| PA4781 F (Rec) | AGCAGCGAATTCC(C)ATGGAGAGCATGCTGGACAGG |
| PA4781 R (Rec) | CCCGGGCTCGAGCAGATGGGTACGCACCCGCG |
|  |  |
| PA2572 Complementation |  |
| C PA2572 F (Rec) (FL) | CCCGGGCTCGAGATGAACGATAGCGCACCTCC |
| C PA2572 R (Rec) | AGCAGCGAATTCCTAGTGGTGGTGGTGGTGGTGCGACTGGCGCAGGGTCAGC |
| C PA2572 F (YN-GYP) | CCCGGGCTCGAGCTCGAATACCTGCATTCCG |
| C PA2572 R (YN-GYP)(FL) (FL) | AGCAGCGAATTCCTAGTGGTGGTGGTGGTGGTGGGTCGTCGACTCCGGGAGGC |

Restriction enzyme cut sites are underlined.

Abbreviations: Primers used to amplify FL (full-length gene), HisK (Histidine kinase domain), Rec (receiver domain), YN-GYP (YN-GYP domain), -TM (gene fragment without the transmembrane domains)

(C) in primer sequence represents additional cystine necessary to clone an in-frame insert into pTRG plasmid.
