# Supplementary material for: An Orphan Chemotaxis Sensor Regulates Virulence and Antibiotic Tolerance in the Human Pathogen Pseudomonas aeruginosa
Source: PLoS One. 2012 Aug 1;7(8):e42205. doi: 10.1371/journal.pone.0042205 (PMC3411652; doi:10.1371/journal.pone.0042205)
Supplement: Table S3 — Genes regulated in the PA2572 mutant compared to the wild-type PAO1strain during exponential growth in LB media (>3.0-fold). (DOCX) [file pone.0042205.s005.docx]

**Table S3**. Genes regulated in the PA2572 mutant compared to the wild-type PAO1strain during exponential growth in LB media (> 3.0-fold).

| Gene | Description | Fold change PAO1/PA2572 | |
| --- | --- | --- | --- |
| PA0283 | *sbp*, sulfate-binding protein precursor | | 4.25 |
| PA0284 | hypothetical protein | | 4.98 |
| PA0547 | probable transcriptional regulator | | 3.13 |
| PA0713 | hypothetical protein | | -3.77 |
| PA0779 | probable ATP-dependent protease | | -3.73 |
| PA0805 | hypothetical protein | | 3.08 |
| PA0806 | hypothetical protein | | 3.54 |
| PA0830 | hypothetical protein | | -4.12 |
| PA0958 | *oprD*, basic amino acid, basic peptide and imipenem outer membrane porin OprD precursor | | -3.24 |
| PA1183 | *dctA*, C4-dicarboxylate transport protein | | 4.52 |
| PA1325 | conserved hypothetical protein | | 9.97 |
| PA1326 | *ilvA2*, threonine dehydratase, biosynthetic | | 11.80 |
| PA1425 | ATP-binding component of ABC transporter | | 4.51 |
| PA1471 | hypothetical protein | | 3.07 |
| PA1559 | hypothetical protein | | 5.33 |
| PA1560 | hypothetical protein | | 4.68 |
| PA1797 | hypothetical protein | | 56.20 |
| PA2018 | *mexY*, Resistance-Nodulation-Cell Division (RND) multidrug efflux transporter | | 11.15 |
| PA2019 | *mexX*, Resistance-Nodulation-Cell Division (RND) multidrug efflux membrane fusion protein precursor | | 16.92 |
| PA2355 | *msuC*, FMNH2-dependent monooxygenase MsuC | | 3.12 |
| PA2357 | *msuE*, NADH-dependent FMN reductase MsuE | | 5.93 |
| PA2358 | hypothetical protein | | 41.25 |
| PA2485 | hypothetical protein | | 3.00 |
| PA2562 | hypothetical protein | | 3.44 |
| PA2655 | hypothetical protein | | 50.03 |
| PA2943 | phospho-2-dehydro-3-deoxyheptonate aldolase | | 3.95 |
| PA3187 | probable ATP-binding component of ABC transporter | | -3.04 |
| PA3190 | probable binding protein component of ABC sugar transporter | | -6.65 |
| PA3432 | hypothetical protein | | -3.18 |
| PA3446 | NAD(P)H-dependent FMN reductase | | 6.22 |
| PA3450 | probable antioxidant protein | | 3.91 |
| PA3530 | conserved hypothetical protein | | 5.09 |
| PA3552 | *arnB*, UDP-4-amino-4-deoxy-L-arabinose--oxoglutarate aminotransferase arnB | | 3.04 |
| PA3553 | *arnC*, undecaprenyl-phosphate 4-deoxy-4-formamido-L-arabinose transferase arnC | | 3.12 |
| PA3555 | *arnD*, undecaprenyl phosphate-alpha-L-ara4FN deformylase arnD | | 3.86 |
| PA3556 | *arnT*, inner membrane 4-amino-4-deoxy-L-arabinose transferase arnT | | 3.84 |
| PA3557 | *arnE*, undecaprenyl phosphate-alpha-L-ara4N flippase subunit ArnE | | 3.99 |
| PA3558 | *arnF*, undecaprenyl phosphate-alpha-L-ara4N flippase subunit ArnF | | 3.23 |
| PA3559 | probable nucleotide sugar dehydrogenase | | 3.15 |
| PA3931 | D-methionine transport system substrate-binding protein, ABC transporter | | 6.19 |
| PA4138 | *tyrS*, tyrosyl-tRNA synthetase | | 5.00 |
| PA4154 | conserved hypothetical protein | | 3.00 |
| PA4200 | hypothetical protein | | -3.82 |
| PA4599 | Resistance-Nodulation-Cell Division (RND) multidrug efflux membrane fusion protein MexC precursor | | 4.45 |
| PA4685 | hypothetical protein | | -14.27 |
| PA4770 | *lctP*, L-lactate permease | | 3.79 |
| PA4771 | *lctD*, L-lactate dehydrogenase | | 3.95 |
| PA4773 | S-adenosylmethionine decarboxylase | | 3.35 |
| PA4774 | hypothetical protein | | 3.60 |
| PA4775 | hypothetical protein | | 3.66 |
| PA4776 | *pmrA*, two-component regulator system response regulator PmrA | | 3.60 |
| PA4777 | *pmrB*, two-component regulator system signal sensor kinase PmrB | | 4.36 |
| PA4781 | cyclic di-GMP phosphodiesterase | | 3.50 |
| PA4782 | hypothetical protein | | 5.10 |
| PA4823 | hypothetical protein | | 6.24 |
| PA4824 | hypothetical protein | | 4.40 |
| PA4825 | *mgtA*, Mg(2+) transport ATPase | | 10.89 |
| PA4826 | hypothetical protein | | 3.73 |
| PA4881 | hypothetical protein | | -3.56 |
| PA5445 | coenzyme A transferase | | 3.67 |
| PA5470 | probable peptide chain release factor | | 7.73 |
| PA5471 | hypothetical protein | | 7.01 |
