# Supplementary material for: An Orphan Chemotaxis Sensor Regulates Virulence and Antibiotic Tolerance in the Human Pathogen Pseudomonas aeruginosa
Source: PLoS One. 2012 Aug 1;7(8):e42205. doi: 10.1371/journal.pone.0042205 (PMC3411652; doi:10.1371/journal.pone.0042205)
Supplement: Table S4 — Genes regulated in the PA2573 mutant compared to the wild-type PAO1strain during exponential growth in LB media (>3.0-fold). (DOCX) [file pone.0042205.s006.docx]

**Table S4**. Genes regulated in the PA2573 mutant compared to the wild-type PAO1strain during exponential growth in LB media (> 3.0-fold).

| Gene | Description | Fold change PAO1/PA2573 | |
| --- | --- | --- | --- |
| PA0024 | *hemF*, coproporphyrinogen III oxidase, aerobic | | -3.05 |
| PA0026 | *plcB*, phospholipase C PlcB | | -5.74 |
| PA0044 | *exoT*, exoenzyme T | | -4.93 |
| PA0141 | conserved hypothetical protein | | -9.38 |
| PA0162 | *opdC*, histidine porin OpdC | | -3.49 |
| PA0164 | probable gamma-glutamyltranspeptidase | | -3.68 |
| PA0169 | hypothetical protein, GGDEF domain protein | | 4.02 |
| PA0170 | hypothetical protein | | 3.81 |
| PA0171 | hypothetical protein | | 3.71 |
| PA0172 | hypothetical protein | | 3.50 |
| PA0200 | hypothetical protein | | -4.57 |
| PA0266 | *gabT*, 4-aminobutyrate aminotransferase GabT | | -3.01 |
| PA0281 | *cysW*, sulfate transport protein CysW | | 3.23 |
| PA0459 | probable ClpA/B protease ATP binding subunit | | -3.99 |
| PA0513 | probable transcriptional regulator | | -3.42 |
| PA0515 | probable transcriptional regulator | | -8.56 |
| PA0516 | *nirF*, heme d1 biosynthesis protein NirF | | -4.22 |
| PA0517 | *nirC*, probable c-type cytochrome precursor | | -14.71 |
| PA0518 | *nirM*, cytochrome c-551 precursor | | -12.52 |
| PA0519 | *nirS*, nitrite reductase precursor | | -13.08 |
| PA0523 | *norC*, nitric-oxide reductase subunit C | | -4.76 |
| PA0524 | *norB*, nitric-oxide reductase subunit B | | -5.48 |
| PA0526 | hypothetical protein | | -4.87 |
| PA0529 | conserved hypothetical protein | | -7.39 |
| PA0603 | probable ATP-binding component of ABC transporter | | -5.88 |
| PA0605 | probable permease of ABC transporter | | -4.60 |
| PA0621 | conserved hypothetical protein | | -3.12 |
| PA0622 | probable bacteriophage protein | | -3.98 |
| PA0633 | hypothetical protein | | -3.23 |
| PA0635 | hypothetical protein | | -3.13 |
| PA0713 | hypothetical protein | | -4.20 |
| PA0830 | hypothetical protein | | -3.77 |
| PA0835 | *pta*, phosphate acetyltransferase | | -5.03 |
| PA0836 | *ackA*, acetate kinase | | -5.49 |
| PA0865 | *hpd*, 4-hydroxyphenylpyruvate dioxygenase | | 6.37 |
| PA0887 | *acsA*, acetyl-coenzyme A synthetase | | 3.45 |
| PA0897 | *aruG*, arginine/ornithine succinyltransferase AII subunit | | -3.33 |
| PA0996 | *pqsA*, probable coenzyme A ligase | | -4.15 |
| PA0997 | *pqsB*, PqsB | | -6.05 |
| PA0998 | *pqsC*, PqsC | | -5.24 |
| PA0999 | *pqsD*, 3-oxoacyl-[acyl-carrier-protein] synthase III | | -4.90 |
| PA1196 | probable transcriptional regulator | | -6.46 |
| PA1197 | hypothetical protein | | -3.39 |
| PA1202 | probable hydrolase | | 8.29 |
| PA1203 | hypothetical protein | | 4.23 |
| PA1325 | conserved hypothetical protein | | 3.81 |
| PA1326 | *ilvA2*, threonine dehydratase | | 5.57 |
| PA1555 | *ccoP2*, cytochrome c oxidase, cbb3-type, CcoP subunit | | -6.59 |
| PA1556 | *ccoO2*, cytochrome c oxidase, cbb3-type, CcoO subunit | | -11.54 |
| PA1557 | *ccoN2*, Cytochrome c oxidase, cbb3-type, CcoN subunit | | -3.86 |
| PA1692 | probable translocation protein type III secretion | | -3.38 |
| PA1694 | *pscQ*, translocation protein type III secretion PscQ | | -3.43 |
| PA1700 | *pcr2*, Pcr2 | | -3.32 |
| PA1701 | *pcr3*, Pcr3 | | -4.65 |
| PA1706 | *pcrV*, type III secretion protein PcrV | | -3.12 |
| PA1707 | *pcrH*, regulatory protein PcrH | | -8.65 |
| PA1708 | *popB*, translocator protein PopB | | -13.00 |
| PA1709 | *popD*, translocator outer membrane protein PopD precursor | | -11.65 |
| PA1710 | *exsC*, ExsC, exoenzyme S synthesis protein C precursor | | -4.35 |
| PA1711 | *exsE*, ExsE | | -7.79 |
| PA1714 | *exsD*, ExsD | | -3.75 |
| PA1716 | *pscC*, type III secretion outer membrane protein PscC precursor | | -3.15 |
| PA1718 | *pscE*, type III export protein PscE | | -30.95 |
| PA1719 | *pscF*, type III export protein PscF | | -5.63 |
| PA1720 | *pscG*, type III export protein PscG | | -3.86 |
| PA1722 | *pscI*, type III export protein PscI | | -3.98 |
| PA1746 | hypothetical protein | | -6.00 |
| PA1789 | hypothetical protein | | -3.09 |
| PA1999 | *dhcA*, DhcA, dehydrocarnitine CoA transferase, subunit A | | 7.34 |
| PA2000 | *dhcB*, DhcB, dehydrocarnitine CoA transferase, subunit B | | 6.28 |
| PA2001 | *atoB*, acetyl-CoA acetyltransferase | | 3.72 |
| PA2007 | *maiA*, maleylacetoacetate isomerase | | 3.93 |
| PA2018 | *mexY*, Resistance-Nodulation-Cell Division (RND) multidrug efflux transporter | | 9.04 |
| PA2019 | *mexX*, Resistance-Nodulation-Cell Division (RND) multidrug efflux membrane fusion protein precursor | | 12.66 |
| PA2026 | conserved hypothetical protein | | -3.88 |
| PA2110 | hypothetical protein | | 5.65 |
| PA2111 | hypothetical protein | | 5.00 |
| PA2112 | conserved hypothetical protein | | 6.16 |
| PA2113 | *opdO*, pyroglutamate porin OpdO | | 5.76 |
| PA2114 | probable major facilitator superfamily (MFS) transporter | | 5.15 |
| PA2116 | conserved hypothetical protein | | 4.44 |
| PA2127 | conserved hypothetical protein | | -3.75 |
| PA2204 | probable binding protein component of ABC transporter | | 4.37 |
| PA2249 | *bkdB*, branched-chain α-keto acid dehydrogenase (lipoamide component) | | 3.09 |
| PA2250 | *lpdV*, lipoamide dehydrogenase-Val | | 3.49 |
| PA2381 | hypothetical protein | | -3.17 |
| PA2430 | conserved hypothetical protein | | 3.02 |
| PA2431 | hypothetical protein, | | 3.96 |
| PA2432 | *bexR*, bistable expression regulator, BexR | | 10.91 |
| PA2433 | hypothetical protein | | 3.10 |
| PA2444 | *glyA2*, serine hydroxymethyltransferase | | -3.83 |
| PA2511 | probable transcriptional regulator | | 3.96 |
| PA2512 | *antA*, anthranilate dioxygenase large subunit | | 10.46 |
| PA2513 | *antB*, anthranilate dioxygenase small subunit | | 29.35 |
| PA2514 | *antC*, anthranilate dioxygenase reductase | | 8.23 |
| PA2663 | *ppyR*, psl and pyoverdine operon regulator PpyR | | -3.33 |
| PA2682 | conserved hypothetical protein | | 4.71 |
| PA2753 | hypothetical protein | | -4.34 |
| PA2754 | conserved hypothetical protein | | -3.76 |
| PA2776 | conserved hypothetical protein | | -3.08 |
| PA2943 | phospho-2-dehydro-3-deoxyheptonate aldolase | | 3.90 |
| PA3038 | probable porin | | 5.29 |
| PA3147 | *wbpJ*, probable glycosyl transferase WbpJ | | -3.04 |
| PA3148 | *wbpI*, UDP-N-acetylglucosamine 2-epimerase WbpI, | | -3.25 |
| PA3188 | probable permease of ABC sugar transporter | | 3.03 |
| PA3234 | probable sodium:solute symporter | | 3.20 |
| PA3235 | conserved hypothetical protein | | 3.09 |
| PA3337 | *rfaD*, ADP-L-glycero-D-mannoheptose 6-epimerase | | -5.68 |
| PA3391 | *nosR*, regulatory protein NosR | | -3.15 |
| PA3392 | *nosZ*, nitrous-oxide reductase precursor | | -4.46 |
| PA3431 | conserved hypothetical protein | | -3.52 |
| PA3432 | hypothetical protein | | -4.55 |
| PA3458 | probable transcriptional regulator | | -3.63 |
| PA3465 | conserved hypothetical protein | | -3.28 |
| PA3530 | conserved hypotheticqal protein | | 4.66 |
| PA3581 | *glpF*, glycerol uptake facilitator protein | | -4.84 |
| PA3584 | *glpD*, glycerol-3-phosphate dehydrogenas | | -8.27 |
| PA3610 | *potD*, polyamine transport protein PotD | | -3.12 |
| PA3613 | hypothetical protein | | -4.16 |
| PA3839 | probable sodium:sulfate symporter | | -4.03 |
| PA3841 | *exoS*, exoenzyme S | | -8.49 |
| PA3842 | *spcS*, specific Pseudomonas chaperone for ExoS, SpcS | | -5.03 |
| PA3876 | *nark2*, nitrite extrusion protein 2 | | -3.09 |
| PA3877 | *nark1*, nitrite extrusion protein 1 | | -9.76 |
| PA3879 | *narL*, two-component response regulator NarL | | -3.58 |
| PA3912 | conserved hypothetical protein | | -3.09 |
| PA3915 | *maoB1*, molybdopterin biosynthetic protein B1 | | -11.53 |
| PA3973 | probable transcriptional regulator | | -4.65 |
| PA4115 | conserved hypothetical protein | | -3.01 |
| PA4138 | *tyrS*, tyrosyl-tRNA synthetase | | 4.17 |
| PA4139 | hypothetical protein | | 5.09 |
| PA4236 | *katA*, catalase | | -4.02 |
| PA4307 | *pctC*, chemotactic transducer PctC | | -3.73 |
| PA4328 | hypothetical protein | | -3.64 |
| PA4352 | hypothetical protein | | -4.31 |
| PA4465 | conserved hypothetical protein | | -3.57 |
| PA4523 | hypothetical protein | | -5.54 |
| PA4526 | *pilB*, type 4 fimbrial biogenesis protein PilB | | -4.16 |
| PA4527 | *pilC*, still frameshift type 4 fimbrial biogenesis protein PilC | | -4.83 |
| PA4528 | *pilD*, type 4 prepilin peptidase PilD | | -4.06 |
| PA4530 | conserved hypothetical protein | | -3.61 |
| PA4571 | probable cytochrome c | | -6.48 |
| PA4587 | *ccpR*, cytochrome c551 peroxidase precursor | | -6.21 |
| PA4610 | hypothetical protein | | -3.83 |
| PA4611 | hypothetical protein | | -8.15 |
| PA4693 | *pssA*, phosphatidylserine synthase | | 3.28 |
| PA4738 | conserved hypothetical protein | | 3.10 |
| PA4747 | *secG*, secretion protein SecG | | 3.22 |
| PA4916 | hypothetical protein | | -3.13 |
| PA4919 | *pncB1*, nicotinate phosphoribosyltransferase | | -3.64 |
| PA5024 | conserved hypothetical protein | | 3.14 |
| PA5035 | *gltD*, glutamate synthase small chain | | 3.73 |
| PA5036 | *gltB*, glutamate synthase large chain precursor | | 3.22 |
| PA5170 | *arcD*, arginine/ornithine antiporter | | -5.84 |
| PA5171 | *arcA*, arginine deiminase | | -19.80 |
| PA5172 | *arcB*, ornithine carbamoyltransferase | | -16.66 |
| PA5173 | *arcC*, carbamate kinase | | -12.72 |
| PA5217 | probable binding protein component of ABC iron transporter | | 3.49 |
| PA5231 | probable ATP-binding/permease fusion ABC transporter | | -3.46 |
| PA5313 | *gabT2*, transaminase | | -3.58 |
| PA5427 | *adhA*, alcohol dehydrogenase | | -4.09 |
| PA5445 | probable coenzyme A transferase | | 4.49 |
| PA5471 | hypothetical protein | | 3.14 |
| PA5494 | hypothetical protein | | -3.51 |
| PA5496 | *nrdJb*, class II (cobalamin-dependent) ribonucleotide-diphosphate reductase subunit, NrdJb | | -3.07 |
| PA5497 | *nrdJa*, class II (cobalamin-dependent) ribonucleotide-diphosphate reductase subunit, NrdJa | | -3.57 |
